# Plasma Concentration of Cortisol Negatively Associates with Platelet Reactivity in Older Subjects

**DOI:** 10.3390/ijms24010717

**Published:** 2022-12-31

**Authors:** Kamil Karolczak, Lucyna Konieczna, Bartlomiej Soltysik, Tomasz Kostka, Piotr Jakub Witas, Joanna Kostanek, Tomasz Baczek, Cezary Watala

**Affiliations:** 1 Department of Haemostatic Disorders, Medical University of Lodz, ul. Mazowiecka 6/8, 92-215 Lodz, Poland; 2 Department of Pharmaceutical Chemistry, Medical University of Gdańsk, ul. Hallera 107, 80-416 Gdańsk, Poland; 3 Department of Geriatrics, Healthy Aging Research Center (HARC), Medical University of Lodz, pl. Hallera 1, 90-647 Lodz, Poland

**Keywords:** cortisol, aging, platelet reactivity, atherosclerosis

## Abstract

The interaction of platelets with steroid hormones is poorly investigated. Age is one of the factors that increase the risk of pathological platelet reactivity and thrombosis. The aim of this study was to assess whether there were associations between platelet reactivity and plasma cortisol levels in volunteers aged 60–65 years. For this purpose, impedance aggregometry in whole blood measured after arachidonic acid, collagen, or ADP stimulation was used to estimate platelet reactivity and mass spectrometry was used to measure peripheral plasma cortisol concentration. Statistically significant negative correlations were observed between cortisol concentration and platelet reactivity in response to arachidonic acid and ADP, but not to collagen. The presented results suggest for the very first time that cortisol is a new endogenous modulator of platelet reactivity in the elderly population.

## 1. Introduction

Blood platelets are the main keepers of undisturbed blood flow in vasculature and blockers of blood lost in the case of damage to blood vessels. Under pathological circumstances, blood platelets are involved in the growth of atherosclerotic plaque on the luminal side of vessels. On these terms, the protective role of platelets is transformed into a harmful or even a life-threatening one [1].

During the process of normal hemostasis, as well as in the course of pathological atherogenesis, blood platelets are exposed to the action of a multitude of factors of exogenous and endogenous origin, affecting platelet activation and reactivity in an inhibitory or an activatory manner [2,3]. Identification of these factors has crucial significance for cardiovascular prophylaxis [4].

In older subpopulations, blood plasma is characterized by higher concentrations of procoagulant molecules being a marker of secretion from platelet granules and platelet activation. Consistently, platelets of older subjects have decreased contents of serotonin. Oxidative modifications of platelet proteins through carbonylation and oxidation of free thiol groups are considered a pivotal alteration of platelet biomacromolecules leading to hyperaggregability. Additionally, the changes in lipid membranes in blood platelets, which gain higher rigidity with aging, are probably responsible for age-associated platelet activation. Blood platelets from older humans exhibit a lesser number of prostacyclin receptors. Undoubtedly, the great contribution to the prothrombotic state of platelets during aging is connected to the significant changes in the endocrine system, especially due to the drop in estradiol synthesis induced by menopause [5]. Possible similar changes due to late-onset hypogonadism in older men are less recognized, however, the role of testosterone in shaping up platelet activation and reactivity in older men and women has been already suggested [6]. It has been proposed that TNF-α can be one of the key players leading to platelet hyperaggregability during aging, due to TNF-α-induced increase in the number of mitochondria in blood platelets and more intense oxygen consumption [7]. Nevertheless, data on the functional state of blood platelets in older subjects are very limited. It is assumed that blood platelets in older people are more prone to activating factors and less sensitive to inhibitors. However, the exact mechanisms leading to these changes are poorly explored and understood [8].

Surprisingly, endocrine hormones, known as pivotal regulators of different body functions, are not commonly regarded as directly involved in the regulation of hemostasis, including platelet function. Such a notion of platelets’ inertness to hormones secreted from endocrine glands also concerns cortisol. Although this glucocorticoid is presently regarded as a cardiovascular hormone, its involvement in atherosclerosis is exclusively noted as shaped out by the cortisol-dependent regulation of glucose and lipid metabolism, together with the induction of obesity and hypertension [9]. Mentioned disturbances are usually associated with abnormal responsiveness to prolonged mental stress, in which cortisol is a biochemical manifestation of psychosocial stressing factors [10].

The most convincing outcomes supporting the presumed involvement of cortisol in atherogenesis derive from patients with Cushing’s syndrome. However, in this disease, cortisol is thought to perpetuate atherogenesis mainly in a way directly dependent on hypertension [11], but not on the activation of blood platelets.

Regrettably, all these studies support only platelet-independent involvement of cortisol in atherogenesis. Concomitantly, they do not emphasize the fact that blood platelets can be hyperactivated by such cortisol-evoked disturbances, such as hypertension [12], obesity [13], hyperglycemia [14,15], or dyslipidemia [16].

In patients with Cushing’s syndrome, an excess of cortisol leads to a set of changes that increase cardiovascular risk, of which hypertension should be mentioned in the first place [17,18]. However, cortisol levels are important for shaping cardiovascular health not only among patients suffering from endocrinological diseases, but also in the general population or among patients with other diseases. Elevated nocturnal cortisol levels in people suffering from hypertension are associated with a greater risk of cardiovascular disease [19]. Hair cortisol has been positively correlated with BMI, which may explain the relationship between cortisol and cardiovascular risk [20]. Based on the conclusions of other studies, it can be suggested that the decrease of cortisol concentration may be a method of cardiovascular prophylaxis, and an element of this prevention may be stress reduction [21]. It is also known that the concentration of cortisol is positively associated with lipid disorders [22].

Repeated reports about the higher prevalence of atherosclerosis under conditions associated with an excess of endo- or exogenous glucocorticoids allow us to raise the hypothesis of a very plausible significant direct involvement of platelets in cortisol-induced atherogenesis, which can be reflected in the associations between plasma cortisolemia and platelet reactivity.

Results published by Moraes et al. [23] encourage looking at blood platelets as cortisol-sensitive objects and, hence, looking at platelet-dependent atherogenesis as possibly modulated in vivo by cortisol in human subjects. It has been shown that blood platelets express subunits of a glucocorticoid receptor, which obviously suggests the responsiveness of blood platelets to the action of cortisol in a receptor-driven manner and indeed Moraes et al. [23] observed that prednisolone (but not dexamethasone) inhibits ADP-induced platelet aggregation and reduces TXB_2_ secretion from platelets [23]. Therefore, the already published results provide a basis for hypothesizing the possibility of direct inhibition of platelet reactivity with cortisol.

However, since no relationship of cortisol levels with platelet responsiveness has been documented in any group of patients prone to the development of atherosclerosis or those with already-confirmed atherosclerosis, the hypothesis based on a significant relationship between the blood concentration of cortisol and the functional state of blood platelets certainly needs further experimental verification.

In the present study we analyzed cortisol levels in the blood plasma of older men and women in order to establish the presumed associations between plasma cortisolemia and markers of platelet reactivity. To the best of our knowledge, this is the very first attempt aiming to evaluate the possible relationships between cortisol and platelet functional state in the population neither affected by any endocrine disease, nor taking glucocorticoids for any medical reason. The results presented herein support the hypothesis that platelet reactivity to arachidonic acid (AA) and adenosine diphosphate (ADP), but not to collagen (COL) remains in a significant dependence on plasma concentrations of cortisol, which implies the cortisol inhibitory action on platelet aggregability in older subjects, and thus appears as a steroid modulating hemostasis.

## 2. Results

### 2.1. General Characteristic of Investigated Subjects

Approximately equal proportions of men and women participated in this study (128 subjects, males/females = 65/63). The mean age was 63 years (±1.7 years), the mean BMI of the whole group was 27.9 kg/m^2^ (±4.6 kg/m^2^), and the mean WHR was equal to 0.93 (±0.09). Nineteen percent of the enrolled subjects were current smokers. There were no other substance abusers among the studied volunteers.

Among the recruited subjects, the representation of diseases was as follows: hypertension—44%, hypercholesterolemia—63%, type 2 diabetes mellitus—9%, chronic heart failure—10%, coronary artery disease—11%, myocardial infarction in the past—0.8%, stroke in the past—2%, joint diseases—42%, osteoporosis—9%, chronic obstructive pulmonary disease—9%, diseases of stomach and duodenum—28%, cancer in the past—5%, ophthalmic diseases—16%, depression—14%. These comorbidities and smoking were used as the confounders for the adjustment of the examined comparisons and associations (adj comorbidities and smoking). All the subjects reported lack of coagulation disorders.

Taken medications reported by subjects were as follows: antithrombotics—3%, antihypertensives—78%, statins—20%, fibrates—3%, antidiabetics—13%, other drugs including supplementation with vitamin D_3_, bisphosphonates, allopurinol, beta mimetics, antihistamines, antidepressants, benzodiazepines, neuroleptics, vinpocetine, trimetazidine, mesalazine, trimebutine, diosmin, and levodopa were taken by less than 2% of participants.

The raw, non-adjusted values of the basic hematological, biochemical, and platelet characteristics of the studied group are given in Table 1.

From the studied group, we excluded the subjects taking antiplatelet drugs (acetylsalicylic acid and/or clopidogrel, ticagrelor) and taking glucocorticosteroids.

### 2.2. Associations between Concentrations of Cortisol in Blood Plasma and Morphological and Biochemical Indices of Atherogenesis with and without Adjustment for Comorbidities

Among morphological indices, plasma levels of cortisol appeared to be significantly negatively associated with the number of blood platelets (PLT; *R*_S_ = −0.213 and *R*_S_LOO_ = −0.213; *p* = 0.02) and plateletcrit (PCT; *R*_S_ = −0.220 and *R*_S_LOO_ = −0.220; *p* = 0.01). Upon adjustment for age, smoking, and comorbidities (see Section 2.1.) these associations were: PLT; *r*_p_adj age, smoking and comorbidities_ = −0.174; *p* = 0.049; *r*_p_adj age, smoking and comorbidities, N = 300_ = −0.171; *p* = 0.003 and PCT; *r*_p_adj adj age, smoking and comorbidities_ = −0.175; *p* = 0.049; *r*_p_adj adj age, smoking and comorbidities, N = 300_ = −0.172; *p* = 0.003.

Among serum biochemical markers of (anti)atherogenesis, the serum level of uric acid was found to show only a statistical tendency regarding the association with concentrations of cortisol and remained beyond a statistical significance (*R*_S_ = 0.171 and *R*_S_LOO_ = 0.171; *p* = 0.055). Additionally, total cholesterol (*R*_S_ = −0.231 and *R*_S_LOO_ = −0.231; *p* = 0.009), HDL- (*R*_S_ = −0.197 and *R*_S_LOO_ = −0.197; *p* = 0.026), and LDL-cholesterol (*R*_S_ = −0.211 and *R*_S_LOO_ = −0.211; *p* = 0.017) were found to be significantly negatively associated with the plasma concentration of cortisol. When adjusted for age, smoking, and comorbidities (see above), the associations for cortisol were: uric acid: *r*_p_adj adj age, smoking and comorbidities, N = 300_ = 0.109, *p* = 0.059, total cholesterol: *r*_p_adj age, smoking and comorbidities_ = −0.137; *p* = 0.018 and LDL-cholesterol: *r*_p_adj adj age, smoking and comorbidities_ = −0.166; *p* = 0.061; *r*_p_adj adj age, smoking and comorbidities, N = 300_ = −0.164; *p* = 0.004.

We have also found a significant positive correlation between the plasma concentration of cortisol and the plasma levels of testosterone (*R*_S_ = 0.314, *R*_S_LOO_ = 0.314; *p* = 0.0003 and *r*_p_adj adj adj age, smoking and comorbidities_ = −0.291; *p* < 0.001) and dihydrotestosterone (*R*_S_ = 0.337, *R*_S_LOO_ = 0.337; *p* = 0.0001 and *r*_p_adj age, smoking and comorbidities_ = −0.293; *p* < 0.001).

### 2.3. Associations between Concentration of Cortisol in Blood Plasma and Platelet Aggregability with and without Adjustment for Comorbidities

In order to assess the in vitro reactivity of blood platelets to the stimulation with physiological agonists, AA, COL, and ADP, we undertook measurements of whole blood aggregation performed with the use of an impedance aggregometry method, which records two parameters of platelet responsiveness to agonists: area under the curve of aggregation (AUC) and maximal value of platelet aggregation (A_max_). These variables, alone or in a combination (AUC*A_max_)/1000), reflect the capability of blood platelets to respond to activating compounds.

We have found that all three parameters of platelet aggregability, measured after stimulation with AA, COL, or ADP, are negatively associated with the concentration of cortisol in blood plasma in a statistically significant manner.

Regardless of whether AUC or A_max_ or their mathematical combination [(AUC*A_max_)/1000] was used as the marker of blood platelet aggregability in response to AA, we noted significant negative associations between the plasma concentration of cortisol and the platelet response: *R*_S_ = −0.235, *R*_S_LOO_ = −0.235, *p* = 0.008, *r*_p_ age, smoking and comorbidities, N = 300_ = −0.170, *p* = 0.003 for [(AUC*A_max_)/1000]_AA_, *R*_S_ = −0.219, *R*_S_LOO_ = −0.219, *p* = 0.015 and *r*_p_adj age, smoking and comorbidities, N = 300_ = −0.121, *p* = 0.036 for AUC_AA_ and *R*_S_ = −0.217, *R*_S_LOO_ = −0.217, *p* = 0.015 and *r*_p_ age, smoking and comorbidities, N = 300_ = −0.155, *p* = −0.008 for A_max AA_.

The same pattern of relationships with plasma concentrations of cortisol was observed in the case of platelet responsiveness to ADP: *R*_S_ = −0.203, *R*_S_LOO_ = −0.203, *p* = 0.025 and *r*_p_ age, smoking and comorbidities, N = 300_ = −0.135, *p* = 0.019 for AUC_ADP_, *R*_S_ = −0.218, *R*_S_LOO_ = −0.218, *p* = 0.015 and *r*_p_ age, smoking and comorbidities, N = 300_ = −0.155, *p* = 0.007 for A_max ADP_, *R*_S_ = −0.220 and *R*_S_LOO_ = −0.220, *p* = 0.015 and *r*_p_ age, smoking and comorbidities, N = 300_ = −0.169, *p* = 0.004 for [AUC*A_max_)/1000]_ADP_. 

Platelet aggregability triggered by COL and reflected by AUC (*R*_S_ = −0.189, *R*_S_LOO_ = −0.189, *p* = 0.04, and *r*_p_ age, smoking and comorbidities, N = 300_ = −0.071, *p* = 0.218) was noted as significantly related to plasma levels of cortisol. In the case of A_max_ (*R*_S_ = −0.168, *R*_S_LOO_ = −0.168, *p* = 0.057 and *r*_p_ age, smoking and comorbidities, N = 300_ = −0.087, *p* = 0.133) and [(AUC*A_max_)/1000] (*R*_S_ = −0.178, *p* = 0.05, and *r*_p_ age, smoking and comorbidities, N = 300_ = −0.072, *p* = 0.211) we noted only a statistical tendency.significance. All these associations for COL were statistically isignificant upon the adjustment for comorbidities.

### 2.4. Comparisons of Platelet Aggregability in Dependence on Cortisol Concentration in Blood Plasma

We divided the whole group of recruited probands into two subgroups. The first group (*n* = 64) included volunteers with a plasma cortisol concentration lower than the median value of cortisol levels calculated for the whole group (*n* = 128, Me = 172.7 ng/mL (IQR: 122.6–222.1 ng/mL)), and the second group (*n* = 64) included volunteers with cortisol plasma levels higher than 172.7 ng/mL. Then we compared the values of markers of platelet reactivity, i.e., AUC, A_max_, and (AUC*Amac/1000), noted during aggregometric measurements triggered with AA, COL, or ADP.

We noted that in the subgroup with the lower cortisol concentration in blood plasma (Me = 122.9 ng/mL; IQR: 108.4–147.9 ng/mL), AUC for AA-induced aggregation was significantly higher than in the subgroup with the higher cortisol plasma concentration (Me = 221.4 ng/mL; IQR: 193.8–270.8 ng/mL). Further, the mean value of A_max_ recorded in the subgroup with lower plasma cortisolemia induced with AA was higher than in those with higher plasma cortisolemia. The combined measure of AA-dependent platelet reactivity (AUC*A_max_/1000) was also higher in the lower cortisolemia-subgroup when compared to the higher cortisolemia-subgroup (Table 2).

For the ADP-triggered aggregation, the same pattern was kept: blood platelets taken from subjects showing a higher plasma concentration of cortisol were less reactive to ADP than those withdrawn from volunteers with a higher cortisol plasma concentration (Table 2).

However, in the case of COL-dependent reactivity, neither comparison showed statistical significance, which stayed in line with the analysis of correlations, where all associations showed border statistical significance for COL (Table 2).

Moreover, we measured the expression of selectin-P and the active form of GPIIb/IIIa on blood platelets activated with AA or COL using flow cytometry. We found no significant correlations between the expressions of either selectin-P or the active form of GPIIb/IIIa on activated blood platelets and the cortisol plasma level (data not shown).

## 3. Discussion

With our highly specific and sensitive method of analysis, we found a median cortisol concentration of 173 ng/mL. In healthy control subjects in the age range of 20–50 years, Rohini et al., 2015 [24] found mean cortisol concentrations (measured with the immunoassay kit in blood samples taken between 9 a.m. and 11 a.m.) of 7.6 µg/mL. Roelfsema et al., 2017 [25] included in their study older subjects (age > 60 y) and reported that, in this age group, the morning cortisol levels (9 a.m.) were in a range between 98–126 ng/mL. Roelfsema et al. used different cortisol assays, like solid-phase RIA, electrochemiluminescence, and chemiluminescent assays. According to our best knowledge, there is no literature data on the specific cortisol concentration range established for the specific age group studied in our experiments. In general, it is considered that the reference range for blood cortisol concentration measured at 8 a.m. is between 51.75–250.1 ng/mL (clevelandclinic.org), which indicates that the median value found by us is within the reference range.

The results presented herein show a negative relationship between the plasma concentration of cortisol and platelet reactivity to AA and ADP. Such an outcome suggests an antiplatelet action of cortisol and, thus, a presumed anti-atherosclerotic action of cortisol. The implication hailed from our data seems to be quite startling since cortisol is generally perceived as a proatherogenic factor. A notion that platelet reactivity correlates positively with plasma concentrations of cortisol would match more glucocorticoid atherosclerotic puzzles and would allow dumping all atherogenic actions, including platelet reactivity, on cortisol action. However, according to us, cortisol is a rather antiplatelet and, thus, antiatherogenic hormone. Completely unestimated remains the balance between the proatherogenic action of cortisol throughdiabetogenicity [14,15] or the increase of concentration of lipids in serum [16], and its antiatherogenic impact due to the presumed inhibition of platelet reactivity. The latter effect is probably direct and may be triggered in blood platelets by cortisol through glucocorticoid receptors present on platelet membranes [23]. 

We noted that the concentration of cortisol associates negatively with platelet number and plateletcrit. These two indices of platelet morphology have already been shown to significantly positively associate with a platelet’s in vitro response to common physiological platelet agonists [6]. These relationships, demonstrated earlier by us contradict the results reported by Beyan et al. [26], who did not reveal correlations between platelet reactivity and any of the morphological indices of blood platelets, including the number of blood platelets. There is, however, some discrepancy in experimental approaches employed for measuring platelet aggregation. Beyan et al. [26] used optical aggregometry, whereas we used impedance aggregometry in whole blood. Thus, our method operates in a more physiological environment, which is whole blood, and not in a platelet-rich plasma or even in isolated platelets suspended in a buffer. On the other hand, optical aggregometry indeed reflects the aggregation of blood platelets, whereas whole blood aggregometry, performed in whole blood, is not a reaction dependent solely on blood platelets but also on all other blood cells, which take part in the formation of a blood clot. Thus, whole blood aggregometry measures some overall aggregation occurring in whole blood with the participation of various blood cellular components, not only blood platelets but also red blood cells and leukocytes.

Although platelet morphometry is still not widely recognized as a predictor of platelet activation or reactivity, according to some authors, it offers a potential use in the estimation of some phenomena related to cardiovascular risks [27]. Taking that into account, cortisol has appeared to be negatively associated with the number of blood platelets, and because PLT and PCT are correlated with platelet reactivity [6], we can speculate that the antiatherogenic action of cortisol may be somehow connected with a decreased rate of thrombopoiesis.

The main analytical method used in the present study was whole blood aggregometry, which can be seriously affected by the number of blood platelets present in the analyzed sample. Apparently, the risk of a significant decrease in platelet aggregation measured with the Multiplate Analyzer in whole blood becomes more probable when the number of blood platelets is below a critical value estimated at 100 × 10^3^/µL [28,29]. In our study, PLT was equal to 213.9 × 10^3^/µL (in the most commonly used reference range, far from the lowest level indicated by Hanke et al. [28] or Stissing et al. [29] as significantly affecting whole blood aggregometry). We have also found that only 6% of our volunteers had PLT lower than 150 × 10^3^/µL (the mean value of PLT calculated for these 8 subjects was 140 × 10^3^/µL, another value far from 100 × 10^3^/µL). Thus, it seems that in our case, there was no serious risk that the reliability of aggregometric measurements undertaken by us could be significantly affected by the low number of blood platelets.

According to our results the concentrations of cortisol correlate negatively with the levels of total-, HDL-, and LDL-cholesterol. Similar results to ours have been reported by Frasier et al. [30], who have announced that a negative correlation between urine cortisol and the blood concentration of HDL cholesterol exists in the general population of men and women younger than the subjects participating in our study (48 and 46 years for men and women, respectively, in the study of Frasier et al. [30] and age between 60 and 65 for both sexes in the present study). Thus, even despite using different biological fluids (urine vs. blood plasma) and measurement techniques (chromatographic measurement of the total concentration of cortisol metabolites, tetrahydrocortisol, allo-tetrahydrocortisol, and tetrahydrocortisone, vs. direct LC/MS measurement of cortisol), the direction of associations between concentrations of HDL cholesterol and cortisol remains invariably negative [30]. Schwertner et al. [31] stated that there is a positive association between the concentration of cholesterol and cortisol and that this association is a characteristic only for patients with coronary artery disease, but not for healthy controls [31]. Such a distinct dichotomy between CAD-affected and CAD-free subjects seems to be quite typical, and similarly, it has been found for cortisol-related steroids, e.g., for aldosterone [32]. It seems pretty difficult to interpret and find common denominators between the outcomes reported by Frasier et al. [30] those reported by Schwertner et al. [31], and our present findings. 

Admittedly, all the mentioned studies included cohorts that could be considered a ‘general group’; however, the general characteristics of each differed significantly. In our study, we analyzed geriatric subjects, who are generally characterized, as older people, by a higher drug load and more extensive medical history. However, the percentage of life-threatening events, like heart infarction or coronary artery disease, remains low (Table 1). Thus, this geriatric group can be referred to as ‘general geriatric’ individuals. Schwertner and colleagues [31] clearly divide their patients into non-CAD and CAD-affected, although a more detailed description of these subjects is impossible due to the lack of published data presenting medical or anthropometric characteristics. Taking into account the negative associations between the concentration of cortisol and the concentration of HDL cholesterol, it appears that cortisol may indeed enhance the risk of atherosclerosis since the HDL fraction of lipoproteins exhibits strong atheroprotective properties. Such a conclusion, however, should not be regarded as a final one as long as some researchers still discover a positive association between cortisol and plasma HDL cholesterol [33].

In our present study, we revealed no significant associations between the plasma concentration of cortisol and fasting glycemia or triglycerides, which remains in fair agreement with the previous report [33].

Our present outcomes may imply some hypothetical mechanism(s) of the indirect inhibitory action of cortisol on platelet reactivity. 

In the previous study, we gave ex vivo and in vitro proof of the antiplatelet action of testosterone and dihydrotestosterone [6]. The antiplatelet action of testosterone and dihydrotestosterone through the modulation of NO synthesis by endothelial cells has also been shown [34], what together implies the possibility that cortisol might influence androgens (we have shown very significant positive associations between cortisol, testosterone, and dihydrotestosterone in the present study), which in turn, may exert their inhibitory effects on blood platelets. If so, the probable pathway of indirect action of cortisol on blood platelets is through regulating the increased levels of antiaggregatory androgens. Such a finding would be in line with a negative correlation between blood platelet function and plasma cortisolemia, as revealed in the present study.

Recently, it has become apparent that anucleated platelets can be affected by various agents in a non-genomic manner [35,36,37]. It can be assumed that the interactions of glucocorticoids with platelets, responsible for the associations observed by us in the current study, may be one of three non-genomic pathways dependent on: (1) the glucocorticoid receptor located in the platelet cytosol, (2) the glucocorticoid receptor located in the platelet membrane, (3) non-receptor (non-specific) interaction of cortisol with the platelet membrane [38]. Both alpha and beta glucocorticoid receptors can be detected in blood platelets at a much higher level in patients with immune thrombocytopenia [39]. The results of Yung et al. [39] make plausible the idea that cortisol inhibits platelet aggregation via its action on glucocorticoid receptors. On the other hand, the lower expression of glucocorticoid receptors in healthy control subjects and the specific augmentation of their expression in immune thrombocytopenia forces us to consider such a possibility as not so obvious and not so easily applicable to older subjects without immune thrombocytopenia. One of the possible downstream mediators of the signal triggered by cortisol in blood platelet glucocorticoid receptors is the protein from Src family kinases (SFKs), which are known to act both as activators and inhibitors of platelet activation and to mediate the rapid signaling of platelet response to different stimuli, including steroids [40].

The glucocorticoid receptor is also present in megakaryocytes and modulates their differentiation [41,42]. This may mean further that the effect of cortisol can be attributed to the earliest stages of platelet production from megakaryocytes.

Elevated platelet counts have already been reported in patients with Cushing’s disease [43,44,45]. Contradictory data can also be found showing a slight decrease in platelet counts in a small percentage of patients with Cushing’s syndrome compared to control volunteers [46]. However, in all the mentioned articles, we can only find a comparison of the number of platelets between the group of patients with Cushing’s syndrome and the control group, with no attempt to estimate the possible relationship between platelet count and the concentration of cortisol. We can speculate that cortisol may be somehow involved in shaping the platelet count in the hypercortisolemic state of Cushing’s syndrome, but the authors of the aforementioned papers have provided no evidence for this. It is also not clear whether these results can be unambiguously related to our outcomes since we obtained results not in the group of patients with endocrine disorders but rather in representatives of the general population of the elderly. The patients described by Sato [44] and Erem [45] were much younger (average age 43 years) than ours. All patients in Erem’s study [45] had pituitary adenomas. In Sato’s study, all participants had adrenal hyperplasia or adenoma. Our volunteers did not report any endocrine disorders. In addition, only women participated in Sato’s study [44]. In both studies [44,45], the numbers of patients were small (10 and 24 patients, respectively). Altogether, it is difficult to relate our results to the few studies published so far.

In another study, also of a small group of volunteers (*n* = 14, overrepresented by women, mean age 48 years) with Cushing’s syndrome secondary to pituitary adenomas, adrenal cortex adenomas, or ectopic production of ACTH, the authors found increased platelet activation as measured by thromboxane B2 levels in the blood. No direct measurements of platelet activation or reactivity have been made [47].

When we leave the articles on endogenous hypercortisolemia aside and turn our attention to exogenous hypercortisolemia, the picture does not become clearer at all. The conclusion from studies evaluating the effect of dexamethasone on platelet activation, summarized in a review by Isidori et al. [48], states that it is currently impossible to clearly assess whether dexamethasone increases or decreases platelet activation. Some studies find glucocorticoid a factor reducing platelet aggregation [49,50], consistent with the results presented by us for cortisol. Others, however, describe dexamethasone as a factor increasing [51,52] or having no significant effect on the functional state of platelets [53,54,55].

It is very important to keep in mind that the associations we found do not necessarily mean cause-effect relationships. Moreover, we found weak negative correlations between platelet reactivity and plasma cortisol levels. Likely, there is no reason to suspect that these correlations should be very high since cortisol per se is not the main hemostatic factor. Nevertheless, the possible cause-effect relationship between platelet reactivity and cortisol should be tested in further experiments in the future.

## 4. Materials and Methods

### 4.1. Chemicals

LC–MS grade, acetonitrile (MeCN), methanol (MeOH), tetrahydrofuran (THF), MS grade formic acid (HCOOH), the analytical standard of cortisol, testosterone, dihydrotestosterone (a minimum purity specification of 99%) and dimethyl sulfoxide were delivered by Sigma (St. Louis, MO, USA). Nitric acid (HNO_3_) was purchased from POCH (Gliwice, Poland). Dichloromethane (DCHM)–HPLC grade was provided by VWR (Radnor, PA, USA). PBS was received from Avantor Performance Materials Poland S.A. (Gliwice, Polska). Arachidonic acid (AA), collagen (COL), and ADP (adenosine diphosphate) were from Chrono-Log Corp. (Havertown, PA, USA).

Ultrapure water was obtained from Milli-Q purification system (Millipore, Bedford, MA, USA). Nitrogen (NM32LA Nitrogen Generator, Peak Scientific Instruments, Billerica, MA, USA) was used as a drying gas.

### 4.2. Study Population

All steps of experiments with the participation of human subjects were undertaken under the guidelines of the Helsinki Declaration for human research. The study was approved by the Committee on the Ethics of Research in Human Experimentation at the Medical University of Lodz. Written abstract of experiment, including detailed information regarding the study objectives, study design, risks, and benefits were presented to each of the volunteers during recruitment to give an opportunity to consider all pros and cons in regard to the participation in the study. Written informed consent was obtained from each individual at the beginning of the experiments. All steps in the recruitment were done according to the protocol used and described earlier [56]. In brief, the research group included 300 subjects (150 men and 150 women), aged 60 to 65 years (group-matched age distribution). For the arm of the study devoted to cortisol, we selected the target population of the final group of volunteers, including 128 participants (63 females and 65 males).

### 4.3. Blood Sampling, Isolation of Blood Plasma, Measurements of Hematological Parameters and Serum Biochemistry

Blood was taken after overnight fasting, always between 8 a.m. and 9 a.m. All of the participants rested for 15 min in a seated position directly prior to blood donation. Blood was collected using aspiration to vacuum tubes (Sarstedt, Nümbrecht, Germany) supplemented with 0.105 mol/l buffered sodium citrate (citrate:blood ratio = 1:9, *v*/*v*), in the case of samples for measurements of platelet aggregation, or with EDTA, in the case of samples taken for analysis of hematological parameters, or without any anticoagulant, in the case of blood samples prepared for serum isolation. In all cases, blood was collected from a peripheral vein cannulated with an 18-gauge needle.

Hematological parameters were measured with the hematological analyzer 5-Diff Sysmex XS-1000i (Sysmex, Kobe, Japan) and serum biochemical parameters were evaluated with the analyzer DIRUI CS 400 (Dirui, Changchun, China).

In order to obtain blood serum, the non-anticoagulated whole blood was incubated for 30 min at 37 °C and centrifuged (2000× *g*/15 min/4 °C). Supernatant (serum) was aspirated and used for further analysis.

Blood plasma was obtained from citrated blood (0.1 mol/L citrate, blood:citrate = 9:1 (*v*/*v*)), centrifuged immediately after blood sampling (1000× *g*/15 min/4 °C), portioned (1 mL), immediately deeply frozen and kept at −80 °C until used further within the following 6 months.

### 4.4. Measurements of Platelet Aggregation and Estimation of Platelet Activation and Reactivity Using Flow Cytometry

Platelet aggregability in whole blood was measured with a Multiplate Analyzer-impedance aggregometer (Dynabyte, Munich, Germany), using citrated blood pre-incubated for 10 min at 37 °C to minimize artefactual platelet activation caused by aspiration. Afterward, 300 µL of blood was mixed with 300 µL of PBS and incubated for 3 min at 37 °C. AA, COL, or ADP was added to achieve the final concentrations of 0.5 mmol/L, 1 µg/mL, or 10 µmol/L, respectively. The recording of aggregation was started immediately after the supplementation with agonists and was tracked for 15 min. Area under the curve (AUC) and maximal aggregation (A_max_) were read as the parameters of platelet aggregability. Aggregometric measurement allowed the characterization of platelet aggregation with a few variables: max aggregation, slope of aggregation curve, the area under the aggregation curve, etc. In our labwe both of these variables are used to characterize platelet reactivity. Those two variables give the optimal measure of the extent of platelet aggregation: how high it is when platelets respond maximally, and how long it lasts before disaggregation starts. According to us, these two measures together are much more reliable measures of the extent of platelet aggregation than just one measure alone. Further, it is more difficult to justify selecting only one of these variables for the final description of aggregation, so we believe that a third variable combining the previous two ([AUC*A_max_)/1000] adds new discriminating power and might be used to get the more complete description of platelet aggregation.

Flow cytometry measurements were performed according to the protocol described earlier [6].

### 4.5. HPLC-MS

Standard solutions of all the analytical steroid standards at the concentrations of 10 µg/mL were prepared by making decimal dilutions. In the first step, 1 mg of steroid standards was dissolved into 1 mL of methanol. Then, 100 µL of this mixture was taken and dissolved into 900 µL of methanol (100 µg/mL). The last step was to take 100 μL of the previous mixture and dilute it to 1 mL with methanol. The resulting concentration of the mixture was 10 µg/mL. The stock standard steroid solutions were stored at −20 °C.

The extraction mixture was prepared by mixing 3 mL of THF with 1.2 mL of DCHM so that the THF: DCHM volumetric ratio was approximately 500:200.

LC–MS analysis was performed on an Agilent 1260 Infinity HPLC system (Agilent Technologies, Santa Clara, CA, USA). An Agilent Infinity HPLC system consists of a binary pump, autosampler, and UV variable wavelength detector coupled to an Agilent 6120 mass detector MS with a quadrupole analyzer (Single Quad, Agilent Technologies, Santa Clara, CA, USA). The chromatographic system coupled to an Agilent 6120 Single Quad mass spectrometer (Agilent Technologies, Santa Clara, CA, USA) was equipped with an electrospray (ESI) ion source operated in positive ion mode.

Chromatographic separation of analytes was carried out on a Poroshell 120 EC-C18 (Agilent Technologies, St. Clara, CA, USA) column with dimensions of 3.0 × 100 mm, 2.7 μm particle size, thermostated at 25 °C. The chromatographic results were collected using ChemStation software B. 04.03 (Agilent Technologies, Santa Clara, CA, USA).

The mobile phase was composed of 0.1% formic acid in Millipore grade water (eluent A) and 0.1% formic acid in LC–MS grade acetonitrile (eluent B). The gradient started linearly from 33.1 to 55.1% B over 12 min, followed by an increase to 100% B over 2 min, remained at a constant level of 100% B for over 2 min, and further decreased to 33.1% over 0.1 min. The column was then re-equilibrated for 3.9 min at initial conditions.

The optimized MS parameters were as follows: ion source temperature of 350 °C, drying gas (N_2_) flow rate of 12 L/min, nebulizer pressure of 60 psig, and capillary voltage of 6000 V. The fragmentor voltage was set individually for each analyzed compound in the range of 100 to 175 V. The MS system was operated in positive ion mode. Selected ion monitoring (SIM) parameters chosen for the quantitative assay development were *m*/*z* 363 for cortisol, 289 for testosterone, 291 for dihydrotestosterone, and 373 and 303 for β methasone and methyltestosterone (internal standards), respectively.

The total run time analysis was 25 min. The injection volume was 5 µL. The flow rate of the mobile phase was set at 0.5 mL/min, the column was thermostated at 25 °C, and the temperature at the autosampler’s storage box was at 4 °C.

Human blood samples were stored at −80 °C. Before analysis, blood samples were thawed at room temperature. An amount of 200 µL of mixture of HNO_3_:H_2_O was added to 200 µL of plasma samples for the deproteinization stage, and the mixture was filled with water to 1 mL. Next, after denaturing proteins, plasma samples were vortexed and centrifuged for 7 min at 7900× *g*. The 980 µL of supernatants were transferred into a 1.5 mL Eppendorf tube and added to 20 µL of two international standards (10 µg/mL of methyl-testosterone solution in methanol and 10 µg/mL of betamethasone solution).

A 700 µL solution mixture to extraction (500 µL of THF, which is the extraction solvent, and 200 µL of DCHM, which is the disperser solvent) was rapidly injected using a Hamilton syringe into 1 mL of plasma sample. The mixture was shaken for about 10 s and then was frozen for 10 min. To separate the phase, the emulsion was centrifuged for 10 min at 5056× *g*. After that, the drop in volume of DCHM (150 ± 10 µL) containing analyzed steroids was collected and evaporated to dryness at 45 °C under vacuum conditions, using a rotary evaporator, a CentriVap (Labconco, Kansas City, MO, USA). The residue was dissolved in 100 µL of methanol, moved to a vial insert, and submitted for LC-MS analysis.

### 4.6. Statistical Analysis

Data are presented as mean ±SD or median and interquartile range (IQR: lower quartile, LQ [25%], to upper quartile, UQ [75%]), depending on the departures of given variables from the normal distribution (verified with Shapiro-Wilk’s). Grubbs’ test and Brown-Forsythe’s test were used to check for the presence of outliers and variance homogeneity, respectively. The bootstrap-boosted ANCOVA was used for the comparisons, adjusted for possible confounders. The bootstrap-boosted partial correlation (*r*_p_) and Spearman’s ranks correlation (*R*_S_) were used to evaluate the associations between variables. The outcomes of the conventional inference tests and simple nonparametric correlation estimates were further validated using the leave-one-out (LOO, jackknife, or *d*-jackknife) techniques. When adjusting the estimated associations for confounding variables (comorbidities) in our multivariate models, we accepted for use as a standard approach the resampling techniques with an adjustment in the sample size to 300 (the size of the overall group enrolled in the present study); it particularly justified the analyses of more complex models that include several variables (explanatory/confounding). In those cases where we present our results obtained after adjusting the sample size to 300, we describe the association as “*r*_p_ age, smoking and comorbidities, N = 300_.” However, in some cases, the adjustment of the sample size to 300 was not necessary because we had found statistically significant associations even with a sample size of 128 (the number of males and females with the complete matrix of the analyzed variables). In that case, we present the association as “*r*_p_adj age, smoking and comorbidities_” (with no indication of N). Differences with *p* ≤ 0.05 were considered statistically significant.

## 5. Conclusions

Different morphological and biochemical factors (with relevance to pro- or antiatherogenic impact), together with platelet reactivity, are specifically related to the plasma concentration of cortisol. This might suggest a multifactorial modulation of the progress of atherogenesis by cortisol. However, our reasoning is merely based on the outcomes of association analyses, and therefore, we certainly need to perform further experiments aimed at figuring out any putative mechanisms standing behind the suggested hypotheses on the impact of cortisol on platelet-derived hemostasis.

## Figures and Tables

**Table 1:** Morphological, biochemical, and platelet function characteristics of investigated group of individuals.

Variable	Mean ± SD or Median (IQR)
Age (years)	63 (61–64)
WBC (10^3^/µL)	5.7 (5.1–6.8)
RBC (10^6^/µL)	4.5 ± 0.35
HCT (%)	39.9 ± 46.3
PLT (10^3^/µL)	213.9 ± 46.3
MPV (µm^3^)	11.3 (10.7–12.0)
PCT (%)	0.24 (0.21–0.27)
PDW (fl)	13.6 (12.3–15.7)
P-LCR (%)	36.1 ± 8.0
Lym (10^3^/µL)	1.9 (1.5–2.3)
Mono (10^3^/µL)	0.5 (0.43–0.62)
Neu (10^3^/µL)	3.1 (2.5–3.8)
Eo (10^3^/µL)	0.1 (0.1–0.2)
Baso (10^3^/µL)	0.02 (0.02–0.03)
Total cholesterol (mg/dL)	209.3 (174.5–241.0)
Triglycerides (mg/dL)	112.2 (82.4–160.5)
Cholesterol HDL (mg/dL)	48.7 (41.6–59.1)
Cholesterol LDL (mg/dL)	132.6 ± 36.9
Glucose (mg/dL)	99.1 (90.8–107.5)
Uric acid (mg/dL)	5.0 ± 1.3
(AUC*A_max_)/1000_AA (a.u.)	320.1 ± 146.4
(AUC*A_max_)/1000_COL (a.u.)	429.5 (306.1–597.6)
(AUC*A_max_)/1000_ADP (a.u.)	297.1 ± 151.6
Homocysteine (µmol/l)	15.1 (12.6–17.7)
Cortisol (ng/mL)	172.7 (122.7–222.1)

Variables are presented as raw non-adjusted values of means ±SD or medians with interquartile ranges; *n* = 128. Abbreviations used: AA, arachidonic acid; ADP, adenosine diphosphate; A_max_, maximal aggregation of blood platelets; AUC, area under aggregation curve; Baso, number of basophils; COL, collagen; Eo, number of eosinophils; HCT, hematocrit; HDL, high-density lipoproteins; LDL, low-density lipoproteins; LYM, number of lymphocytes; Mono, number of monocytes; MPV, mean platelet volume; Neu, number of neutrophils; PCT, plateletcrit; PDW, platelet distribution width; P-LCR, platelet-large cells ratio; PLT, platelet count; RBC, red blood cell count; WBC, white blood cell count.

**Table 2:** Reactivity of blood platelets in dependence on plasma concentration of cortisol.

	Lower Cortisol Concentration (*n* = 64)			Higher Cortisol Concentration (*n* = 64)		
	AUC	A_max_	(AUC*A_max_)/1000	AUC	A_max_	(AUC*A_max_)/1000
AA	2630.7 ± 534.7	134.3 (113.7; 153.3)	346.3 ± 141.2	2410.5 ± 683.3 ^#^	124.3 (99.8; 141.9) ^n.s.^	294.8 ± 149.1 ^#^
COL	2898.8 ± 880.6	157.6 ± 42.3	451.7 (317.8; 616.1)	2706.1 ± 828.5 ^n.s.^	148.4 ± 42.7 ^n.s.^	395.5 (277.6; 582.7) ^n.s.^
ADP	2451.8 ± 652.8	127.4 ± 30.5	324.2 ± 160.9	2215.8 ± 671.1 ^#^	115.4 ± 32.8 ^#^	270.1 ± 139.1 ^#^

Variables presented as means ±SD, median with interquartile ranges (from lower [25%] to upper [75%] quartile) without and upon adjustment for comorbidities and smoking; *n* = 64. Comparisons between markers of platelet reactivity found in the subgroups with lower and higher blood plasma cortisolemia made with the use of the bootstrap-boosted ANCOVA with the adjustment for age, smoking, and comorbidities. Reactivity of blood platelets was measured with impedance aggregometry (see ‘*Materials and methods*’) in response to arachidonic acid (AA), collagen (COL), or ADP (adenosine diphosphate) and recorded as an area under aggregation curve (AUC) or a maximal aggregation (A_max_). These variables were used to calculate (AUC*A_max_)/1000. Differences with a statistical significance of at least *p* < 0.05 are marked with #. * denotes the boundary significance (*p* = 0.058) estimated with the bootstrap-boosted ANCOVA after the adjustment for age, smoking, and comorbidities and n.s. denotes lack of statistical significance.

## Data Availability

The data presented in this study are available on request from the corresponding author. The data are not publicly available due to ethical reasons.
